# Weight Status, Physical Fitness, and Health-Related Quality of Life among Chinese Adolescents: A Cross-Sectional Study

**DOI:** 10.3390/ijerph16132271

**Published:** 2019-06-27

**Authors:** Xiangren Yi, You Fu, Ryan Burns, Meng Ding

**Affiliations:** 1 Department of Sport and Health, School of Physical Education, Shandong University, Jinan 250061, China; 2 School of Community Health Sciences, University of Nevada, Reno 1664 N. Virginia Street, Reno, NV 89557, USA; 3 Department of Health, Kinesiology, and Recreation, University of Utah, 6951 Wellwood Road, 1Y Midvale, Salt Lake City, UT 84047, USA; 4 School of Physical Education, Shandong Normal University, Jinan 250061, China

**Keywords:** cardiorespiratory fitness, musculoskeletal fitness, obesity, health outcomes

## Abstract

Purpose: The impact of physical fitness (PF) on adolescents’ health-related quality of life (HRQOL) is an important health issue in China. The purpose of this study was to identify whether body mass index (BMI), cardiorespiratory fitness (CRF), and musculoskeletal fitness (MSF) influences HRQOL among Chinese adolescents. Method: The participants were 10,007 students (boys = 5276, 14.14 years ± 1.79; girls = 4829, 14.22 years ± 1.81) who were randomly selected from 30 secondary schools in Shandong, China. BMI, CRF, MSF, and HRQOL were measured and analyzed using ANCOVA and multiple regression. Results: BMI and physical fitness variables were partially associated with HRQOL in Chinese adolescents. ANCOVA showed a significant difference among BMI categories in terms of physical sense (PS), living convenience, and self-satisfaction (SS) for boys, but this difference was only seen with social activity opportunity (SAO) for girls. Multiple regression found that BMI was significantly associated with SAO. For boys, CRF was associated with the teacher and student relationship and SS, whereas MSF was only associated with PS. For girls, CRF was significantly linked with the parent and children relationship, learning capacity, and attitudes and self-perception (S-P), while MSF was associated with S-P. Conclusion: Enforcing physical activity and enhancing PF will be a crucial pathway in improving adolescents’ HRQOL in China.

## 1. Introduction

The high prevalence of overweightness and obesity among children and adolescents has been a large public health concern for the past three decades in China. A national survey on Students’ Physical Fitness and Health in 2010 reported that the obesity rate of urban boys, urban girls, countryside boys, and countryside girls (7–22 years) were 13.33%, 5.64%, 7.83%, and 3.78% respectively. The overweight rate of urban boys, urban girls, countryside boys, and countryside girls (7–22 years) were 14.81%, 9.92%, 10.79%, and 8.03%, respectively, which have increased by 1.94%, 0.63%, 2.76%, and 1.15%, respectively, since 2005 [1]. The Nutrition and Chronic Disease Status of Chinese Residents (2015) also revealed that 9.6% of the population is obese and 6.4% can be classified as overweight among 6–17 year-old Chinese children and adolescents, increasing by 5.1% and 4.3%, respectively, since 2002 [2]. Relative to developed countries, China’s prevalence of overweightness and obesity is relatively low, but this nevertheless has progressively increased over the past decades.

The rising prevalence of overweightness and obesity has correlated with a concomitant decrease in cardiorespiratory fitness (CRF) and musculoskeletal fitness (MSF), which may ultimately attenuate health-related quality of life (HRQOL) [3,4]. It has been documented that HRQOL is inversely associated with weight gain and the risk of overweightness and obesity in children and adolescents [5]. Previous studies have identified factors that may influence HRQOL and analyzed the association between overweightness and obesity and HRQOL. For instance, literature indicated that overweightness and obesity impairs HRQOL in children, especially those in the greatest compromised physical and social dimensions [6,7,8] and those with emotional or mental issues and/or self-esteem and behavioral problems [7,9,10]. This adds concern regarding the impact of obesity-related complications and comorbidity on self-esteem and HRQOL [11].

Specifically, Cameron and colleagues found that a lower HROQL may be a predictor, particularly related to psychosocial well-being, indicating a bi-directional association between obesity and HROQL [12]. Conversely, a recent study showed that overweightness and obesity are not significantly associated with HROQL or socio-economic circumstances; socio-cultural factors should be considered to help elucidate such relationships [13]. The impact of overweightness and obesity on HRQOL may differ among cultures [14], ages [15,16], genders [15,17], incomes [18,19], lifestyles, and ethnicities [20].

Extensive literature has revealed that CRF and MSF are significantly associated with serious health consequences, such as cardiovascular disease (CVD), diabetes, and various cancers [21,22,23,24], independently predicting a higher threat of CVD and mortality across various ages. Higher levels of physical fitness have been shown to be associated with higher levels of HRQOL in older adults and chronically diseased populations [25,26,27].

Recognized as one of the most important health markers, excellent physical fitness in adolescence yields more positive health outcomes [28,29] and highlights the demand to reinforce physical activity in educational monitoring systems.

Although some studies have examined the relationship between CRF and HRQOL [30,31] and the relationship between overweightness and obesity and cardiorespiratory fitness in children and adolescents [29], only two studies with a small sample size have examined kindergarteners’ perceptions related to quality of life across different weight statuses in Hong Kong [32] and the influence of youth overweightness and obesity on quality of life in mainland China [33]. Little is known about the relationship between physical fitness, BMI, and HRQOL as they interact among Chinese adolescents. Therefore, the hypothesis of the present study is that the weight status and physical fitness influence HRQOL. The purpose of this study was to examine the associations among CRF, MSF, and BMI on HRQOL in Chinese adolescents and to identify whether HRQOL is statistically different among BMI, CRF, and MSF categories. This study further explores the association of youth overweightness/obesity with HRQL, self-esteem, and bullying, as well as the association of physical fitness with physical, emotional, and social health and functioning. Knowledge about these relationships may facilitate the development of specific, individually tailored physical activities in a more appropriate manner.

## 2. Materials and Methods

### 2.1. Study Setting and Participants

Using cross-sectional research design, the present study was conducted during the 2016 to 2017 academic year in Shandong Province, China. The province comprises [25] 17 regions with different geographical and demographic characteristics. The participants were recruited from 30 secondary schools (20 middle schools and 10 high schools) in 10 regions. In each region, one urban middle school, one rural middle school, and one urban high school were randomly selected with the purpose of ensuring geographical and cultural diversity. Schools with an enrollment below 300 students were excluded in this study. Initial participants consisted of 10,680 students (aged 12–18 years). Prior to data analysis, 673 students were excluded due to the absence on one of the physical fitness tests. The final participants were 10,007 students, including 5210 boys (52.1%; 14.14 ± 1.79 years) and 4797 girls (47.9%; 14.22 ± 1.81 years). The research was approved by the ethical committees of Shandong University, China.

### 2.2. Procedure

There were 10 superintendents in each region who supervised the evaluators in the schools. A total of 60 evaluators were recruited from middle and high school physical education teachers who had experiences in the National Student Fitness Test Program and adolescent physical fitness evaluation. All evaluators were trained with a testing manual illustrating all the test guidelines, procedures and protocols. In addition, to standardize and homogenize the method of assessment and quality control, all evaluators completed two seminars in order to enhance intra- and inter-tester reliability. All evaluators organized students to measure physical fitness and guided students to answer online questionnaires. Six graduate students operated computers to collect online survey data from each school.

Evaluators administered all tests following standard operating procedures. The participants completed a standardized form, which included socio-demographic data such as region, school, age, gender, date of birth, grade, and ethnicity.

### 2.3. Measures

BMI: Weights and heights of barefooted students in light clothing were measured with a digital electronic weighing scale (HW-VB900, lEJIA, China) and recorded to the nearest 0.1 kilogram. BMI was computed by utilizing weight divided by the square of students’ height (BMI = weight (kg)/height (m)^2^). Participants were categorized into obese, overweight, normal weight, and underweight based on the international BMI cutoff values.

Physical fitness: Physical fitness was assessed employing National Students’ Physical Fitness and Health 2014 (NSPFH 2014) [34], which contained five tests that gauged different components of fitness: BMI, sit and reach (flexibility), standing-long jump (explosive strength), sit-ups for girls (abdominal strength and endurance), pull ups for boys (upper body strength and endurance), and a 1000 m run for boys or 800 m run for girls (cardiorespiratory endurance). These test items are reliable and valid instruments for measuring adolescents’ physical fitness in China.

CRF was assessed by a 1000 m/boy and girl 800m/girl run held during physical education classes in ] middle and high schools. The 1000 and 800 m runs were measured once to evaluate students’ CRF. Students were required to maintain a steady pace and complete the task as quickly as possible. Safety and quality control during the measurements were highlighted. Participant performances in the CRF test were classified into one of four levels in terms of NSPFH 2014 in China. The participants with extreme CRF performances were classified either as performing poorly (i.e., a score of less than 60) or performing very well (i.e., a score of more than 90).

The main health-related MSF components are maximal strength (isometric and dynamic), explosive strength, endurance strength, and isokinetic strength. MSF was assessed by a standing-long jump test and pull ups/sit ups. In this study, we only employed the standing long jump test because many schools did not report the data of the pull up test. The standing broad jump test has been widely utilized for assessing explosive strength.

Health-related quality of life: HRQOL was measured using the Quality of Life Scale for Children and Adolescents (QLSCA), [35] a self-reported, generic measure of HRQOL validated for children and adolescents aged 7–18 years old. The QLSCA measures HRQOL in 13 dimensions: teacher and student relationship, student partnership, parent and children relationship, learning capability and attitude, self-perception, physical sense, negative moods and emotions, homework attitude, life convenience, social activities, sport capacity, self-satisfaction and others. The QLSCA items employed a five-point Likert-type scale to assess either frequency or intensity. The recall period was two weeks. Scores for each dimension were calculated and higher scores indicated better HRQOL. The Chinese version of the QLSCA has been found to have acceptable levels of reliability and validity [35].

### 2.4. Statistical Analyses

Descriptive statistics for gender, BMI, and CRF and MSF were calculated to characterize participants. The grade level for each student was coded as middle school (grades 6–8) or high school (grades 9–12). Both CRF and MSF were categorized using a score (poor = <60, fair = 60–74, good = 75–89, and excellent = ≥90). The correlation analyses were performed to verify the potential linear associations among demographic characteristics, fitness, BMI, and quality of life. Analysis of covariance (ANCOVA) was performed with co-variable age to analyze differences of the QLSCA dimensions among categories of CRF, MSF, and BMI by gender. Multiple linear regression models were estimated to determine associations between physical fitness, BMI, and quality of life, with QLSCA dimensions as independent variables and age, gender, CRF and MFI, and BMI as dependent variables. Lastly, models with BMI, CRF, and MFI were employed to analyze the independent effect of each variable. All values were expressed as the mean ± standard deviation (M ± SD) or frequency analysis numbers (percentage). The significance level was set at *p* ≤ 0.05. All analyses were performed using SPSS 23.0 (IBM Corp., Armonk, NY, USA).

## 3. Results

The descriptive statistics for gender groups are presented in Table 1. There was a higher percentage of overweightness or obesity in boys (19.4%) than in girls (18.9%). The mean CRF and MSF was 4.27 ± 0.89 and 4.22 ± 0.89, respectively, for the boys and 18.29 ± 34.19 and 186.40 ± 33.30, respectively, for the girls. Most of the items for each of the dimensions of QLSCA are similar between genders, except for self perception, social activity opportunity, sport capacity, and mean score, which are higher among boys.

To examine the differences of QLSCA values amongst the different BMI, CRF, and MSF categories, ANCOVA was performed to verify whether QLSCA was worse in students with overweight/obesity and better in students with higher levels of physical fitness (Table 2). However, only several dimensions showed significant differences in this test. In boys, significant differences among BMI categories were found in physical sense (F (3,5204) = 3.542, *p* = 0.014, *η^2^*= 0.002), living convenience (F (3,5204) = 4.462, *p* = 0.005, *η^2^* = 0.003), and self-satisfaction (F(3,5204) = 3.498, *p* = 0.015, *η^2^* = 0.002). In girls, significant differences in BMI were observed with different teacher and student relationships and social activity opportunities in this study. The parent and children relationship in boys (F(3,5204) = 2.310, *p* = 0.042, *η^2^* = 0.002) and girls (F(3,5204) = 1.786, *p* = 0.013, *η^2^* = 0.008) are significantly different among levels of CRF. In boys, several dimensions showed significant differences in terms of MSF level, teacher and student relationship (F(3,5204) = 4.384, *p* = 0.002, *η^2^* = 0.003), student partnership (F(3,5204) = 3.117 *p* = 0.014, *η^2^* = 0.002), parent and children relationship (F(3,5204) = 4.195, *p* = 0.002, *η^2^* = 0.003), physical sense (F(3,5204) = 2.805, *p* = 0.024, *η^2^* = 0.002), and negative moods and emotions (F(3,5204) = 2.435, *p* = 0.045, *η^2^* = 0.002). The exception is student partnership (F(3,5204) = 4.019, *p* = 0.003, *η^2^* = 0.003) in girls.

BMI, CRF, and MSF were utilized as predictors of health-related quality of life (Table 3). The multiple regression model (1) controlling for age showed that BMI was significantly associated with social activity opportunities in boys (β = −0.040, *p* = 0.019) and girls (β = −0.059, *p* = 0.000). In boys, CRF was significantly associated with the teacher and student relationship (β = −0.080, *p* = 0.000) and self-satisfaction (β = 0.064, *p* = 0.002), but MSF was only associated with physical sense (β = −0.080, *p* = 0.000). In girls, CRF was significantly linked with negative moods and emotions, physical sense, and the parent and children relationship, whereas MSF was linked with social activity opportunities and self-perception. The multiple regression model (2) demonstrated that the results with BMI are the same as those of the model (1) variables. In girls, CRF was significantly associated with self-perception, learning capacity and attitudes, and homework attitude, except for model (1) variables. MSF is associated with the teacher and student relationship in boys (β = −0.034, *p* = 0.013) and student partnership (β = −0.042, *p* = 0.016) in girls, except for model (1) variables.

## 4. Discussion

The purpose of the present study is to examine the impact of overweightness/obesity and physical fitness on the health-related quality of life in Chinese adolescents. A secondary purpose was to assess whether Chinese adolescents’ BMI, CRF, and MSF can predict their HRQOL. The results suggested that BMI and CRF were associated with Chinese adolescents’ HRQOL, which is in line with the hypothesis of this study. Results also indicated that the level of the HRQOL was generally low, and a lower level of HRQOL was found in overweight and obese individuals. Most of the dimensions of QLSCA showed no statistically significant differences in this study.

From overweightness and obesity perspectives, previous studies presented evidence that overweightness/obesity were negatively associated with several aspects of adults’ HRQOL [36] and, thus, could impact the RQOL of children and adolescents, mainly in social, emotional, school, and physical dimensions of HRQOL [10,37,38]. In accordance with those findings, our study suggested that there were significant differences in terms of physical sense, living convenience, and self-satisfaction among boys with different weight status categories. In girls, there were significant differences in teacher and student relationships and social activity opportunities, however, no other dimensions of HRQOL were influenced significantly by BMI categories. A multiple regression analysis was employed to further analyze the impact of BMI on quality of life. The results showed that weight status significantly influenced social activity opportunity in both boys and girls.

The results are also partially consistent with those of a European KIDSCREEN research [39]. However, some studies did not find the effect of body weight on HRQOL. For instance, Kruger et al reported that their study did not reveal a significant relationship between BMI and QOL [40]. A higher BMI level was not associated with quality of life, but body weight increased with declining physical fitness and health conditions [41]. BMI and body fat were significantly associated with sports practice and good quality of sleep [42]. The results from different studies may illustrate that comparisons were different between BMI categories because overweightness and obesity were combined into a single category. Furthermore, it is possible these results were affected by differences in sample size, gender, age, and the range of BMI used.

No studies so far have utilized QLSCA to evaluate the association between CRF and HRQOL in large population-based samples of adolescents in China. Our study revealed that CRF is associated with the dimensions of the teacher and student relationship and self-satisfaction in boys; the CRF in girls was associated with the dimensions of the parent and children relationship, learning capacity and attitudes, and self-perception. These findings support our beliefs that teacher and parent support and concern are essential components that could affect adolescents’ HRQOL. These results differed with previous studies, but it is reasonable to suggest that teacher and parent support can significantly influence participation in school-based physical activity. In China, high school teachers and parents do not encourage students to participate in physical activities because they believe that physical activity participation will sacrifice time for study thus affect academic performance. Meanwhile, most secondary school teachers do not pay attention to physical activity but emphasize students’ academic performance due to the school requirements on academic achievement and competitiveness amongst schools [43,44], even though there are studies that show that higher levels of physical fitness are beneficial to academic performance [45,46]. These may be plausible explanations as to why learning capacity and attitudes are more closely associated with HRQOL in girls.

It has been documented that CRF was positively associated with both physical well-being and emotional components of HRQOL in adults [47,48]. Improvements in CRF can lead to positive change on the mental health of children and adolescents [29]. Morales et al. [49] reported CRF is associated with physical well-being and social support and peers among boys and is associated physical well-being, self-perception, and social acceptance in girls. A positive association existed between CRF and waist-to-height, resting heart rate, and moderate to vigorous physical activity MVPA [50]. In this study, our results are partially consistent with social support in boys and self-perception in girls. However, a few researchers examined the associations between CRF and HRQOL, which warrants further study in this area.

Some studies found that MSF was associated with social acceptance and social support [44] and could promote the general health, psychological, and physical components of HRQOL [41]. The present study revealed that the association between the MSF and HRQOL were particularly strong for the physical sense and teacher and student relationship in boys, as well as student partnership and self perception in girls. This result is partially consistent with the findings of previous studies that observed associations between MSF and physical HRQOL dimensions, but has not observed associations between MSF and mental health dimensions [26,48]. This result demonstrated that MSF has a slight impact on the HRQOL of girls and boys. However, the present study exposed gender differences in HRQOL dimensions that are linked with MSF because it is probably more important for girls in psychological and emotional dimensions [17]. Gender differences in the impact of MSF on HRQOL could be attributed to differences in needs of explosive and endurance strength, which require exercises and sports in which adolescents participate in all the dimensions.

This study examined the associations between BMI, CRF, and MSF, and HRQOL. In boys—with BMI, CRF, and MSF in the model, while controlling for age—the association of CRF or overweightness with the HRQOL was stronger than that of MSF. CRF was more closely associated with HRQOL than BMI and MSF in girls. The results showed that, in girls, CRF has a significant impact on physical sense, negative moods, and emotion and self-perception. This study supported previous findings that low levels of physical fitness in children and adolescents results in reduced self-esteem [45,46], increased depression [51,52] and anxiousness [53,54], and lower HRQOL for the bullying dimension [39]. Sloan and Sawada (2009) verified the associations between CRF and HRQOL in young males and found a positive relationship between fitness levels and mental and physical components of HRQOL. One study analyzed the relationship of overweightness/obesity, CRF, and MSF on HRQOL in older people. It found that physical fitness affected physical health HRQOL subscales [26].

Our study found that both boys’ and girls’ BMIs were significantly associated with social activity component of HRQOL, which illustrated that overweightness and obesity may hamper their social activity and further affect education, learning, and working opportunities in China. CRF and MSF are crucial parts of HRQOL, however, we believe that learning capacity and achievement are more important components affecting student HRQOL than physical fitness levels in China.

This study has several strengths. First, it is the seminal study using the QLSCA that assessed the influences of PF and BMI on Chinese adolescents’ HRQOL, exploring important but relatively unexplored Chinese adolescent HRQOL issues. Second, this study was established on a well-verified instrument, thus contributing to the reliability and validity of this approach in the context of physical fitness and QLSCA for Chinese students. Third, this study employed a random, large, and population-based sample of 80 public schools to elucidate the influences of BMI and physical fitness indictors associated with HRQOL among Chinese adolescents. Random large sampling allows us to better determine mean values and outliers, provide required precision, and avoid errors in this study. Finally, this study is a combination of self-reports and objective measures that involves various domains of CRF, MSF, and QLSCA.

This study has some limitations that may influence the generalizability of its outcome. First, this study utilized a cross-sectional design that prevents us from making causal inference. Future longitudinal research is needed to better understand how BMI and physical fitness affects HRQOL over time. Second, generalizations could be limited based on our outcome because of the use of the QLSCA in this study, which is deficient in accounting for chronic illness or comorbidities that are significantly associated with both overweightness/obesity, physical fitness, and HRQOL. Finally, it is noticed that epigenetic influences on both overweightness/obesity and physical fitness cannot be controlled in this study.

## 5. Conclusions

This study provides an initial approach using the Chinese version of QLSCA to examine the influences of physical fitness and BMI on Chinese adolescents’ HRQOL. The findings of this study showed that overweightness and obesity significantly influence social activities and self-perception dimensions of HRQOL, but CRF and MSF are significantly associated with some dimensions of HRQOL. This study partially supports previous research using the European HRQOL. The importance of physical fitness was still supported by our findings that most of the students with good and excellent levels of physical fitness had a higher level of HRQOL. Our results suggest that exercise programs need to be well-designed by school administration in terms of educational systems, supervising overweightness and obesity, enforcing physical activity, and enhancing levels of physical fitness, which could be effective in improving adolescents’ HRQOL in China.

## Figures and Tables

**Table 1:** Descriptive Characteristics and Comparison.

Variable	Boy n = 5276	Girl n = 4829	*p*
Age (years)	14.14 (1.79)	14.22 (1.81)	0.459
Height (cm)	165.35 (9.24)	161.90 (8.40)	0.043
Weight (kg)	56.12/13.70	55.63/12.61	0.025
BMI (kg/m^2^)	20.44/4.45	20.29/3.78	0.481
Overweightness/ obesity (/%)	19.4	18.9	0.435
Cardiorespiratory fitness (1000/800 m)	4.27/0.89	4.22/0.89	0.006
Standing broad jump	187.29/34.19	186.40/33.30	0.001
QLSCA dimensions			
Teacher and student relationship	146.33 (37.16)	146.40 (36.40)	0.931
Student partnership	159.59 (34.40)	159.63 (33.98)	0.955
Parent and children relationship	143.93 (38.50)	143.59 (38.44)	0.658
Learning capacity and attitudes	129.12 (43.40)	127.62 (43.06)	0.083
Self-perception	125.03 (42.39)	123.31 (41.42)	0.040
Physical sense	136.34 (40.17)	136.34 (38.75)	0.994
Negative moods and emotion	133.90 (38.84)	132.91 (38.61)	0.202
Homework attitudes	137.63 (41.28)	138.46 (40.76)	0.312
Life convenience	160.79 (39.96)	161.08 (39.48)	0.319
Social activity opportunity	130.98 (40.61)	129.14 (39.44)	0.022
Sport capability	140.94 (39.31)	137.08 (38.61)	0.000
Self-satisfaction	154.09 (39.14)	152.80 (52.13)	0.159
Other	144.08 (33.99)	144.04 (34.88)	0.951

**Table 2:** Analysis of Covariance of BMI, CRF, and MSF.

Boy							
BMI	UW	NW	OW	OB	F	*p*	*η^2^*
PS	137.84/40.52	135.05/40.01	134.10/39.67	138.73/39.89	3.542	0.014	0.002
LC	160.18/39.72	159.84/40.80	161.09/39.37	166.45/37.10	4.462	0.004	0.003
SS	154.92/38.72	153.01/39.85	154.56/38.32	158.82/37.82	3.498	0.015	0.002
OT	144.33/33.87	142.94/34.32	144.96/34.35	147.38/32.49	2.858	0.036	0.002
CRF	Poor	Fair	Good	Excellent			
TSR	147.87/37.35	148.01/36.90	145.04/37.53	143.36/36.59	3.17	0.007	0.003
PCR	145.45/37.57	145.00/39.05	142.96/36.68	141.50/37.97	2.31	0.042	0.002
SAO	131.52/40.15	130.55/40.97	130.36/40.12	131.54/40.92	3.657	0.004	0.003
MSF	Poor	Fair	Good	Excellent			
TSR	148.96 (36.66)	143.95 (38.27)	146.82 (26.10)	148.42 (36.54)	4.384	0.002	0.003
SP	161.18 (32.58)	158.03 (35.55)	158.95 (34.72)	162.62 (32.65)	3.117	0.014	0.002
PCR	147.18 (37.64)	141.44 (39.78)	144.48 (37.26)	145.51 (38.19)	4.195	0.002	0.003
PS	133.05 (42.18)	136.32 (40.42)	136.51 (39.27)	139.01 (39.13)	2.805	0.024	0.002
NME	130.66 (39.12)	134.15 (38.98)	133.81 (38.42)	136.33 (38.80)	2.435	0.045	0.002
Girl							
BMI	UW	NW	OW	OB			
TSR	147.07/36.95	146.86/35.61	146.77/35.98	142.21/37.95	2.076	0.004	0.007
SAO	129.63/40.97	130.58/38.16	125.31/38.58	124.31/39.93	3.504	0.007	0.003
CRF	Poor	Fair	Good	Excellent			
PCR	145.55/37.22	145.55/37.22	142.38(38.28)	141.29 (40.13)	1.786	0.013	0.008
LCA	128.53/43.66	127.87/42.92	126.84/37.79	124.28/43.86	1.629	0.032	0.007
S-P	123.93/42.43	123.34/40.79	124.81/37.65	120.22/43.16	2.173	0.001	0.01
OT	143.97/37.85	145.09/33.11	139.32/39.01	143.16/34.61	1.64	0.03	0.008
MSF	Poor	Fair	Good	Excellent			
SP	157.37 (36.07)	159.15 (33.65)	160.29 (33.43)	161.68 (33.25)	4.019	0.003	0.003

TSR: teacher and student relationship; SP: student partnership; PCR: parent and children relationship; LCA: learning capability and attitude; S-P: self-perception; PS: physical sense; NME: negative moods and emotions; HA: homework attitude; LC: life convenience; SAO: social activities opportunity; SC: sport capacity; SS: self-satisfaction; OT: other.

**Table 3:** Relationships between BMI, Physical Fitness, and Quality of Life.

Model 1									
	Boys					Girl			
	B	β	P			B	β	P	
BMI									
LC	0.001	0.063	0.000	*F =* 5.464	SAO	−0.001	−0.059	0.000	*F* = 4.742
SAO	−0.001	−0.040	0.019	*R^2^* = 0.003	SS	0.001	0.036	0.029	*R^2^* = 0.003
CRF									
TSR	−002	−0.080	0.000	*F* = 7.384	NME	0.006	0.114	0.000	*F* = 9.759
SS	0.002	0.064	0.002	*R^2^* = 0.005	PS	−0.004	−0.076	0.000	*R^2^* = 0.007
					PCR	0.003	0.047	0.002	
MSF									
PS	0.001	0.040	0.004	*F* = 8.236	SAO	0.001	0.042	0.010	*F* = 5.222
				*R^2^* = 0.002	S-P	0.001	0.037	0.022	*R^2^* = 0.004
Model 2									
	Boys					Girl			
	B	β	P			B	β	P	
BMI									
LC	0.001	0.060	0.000	*F =* 5.151	SAO	−0.001	−0.052	0.002	*F* = 4.991
SAO	0.000	-0.039	0.023	*R^2^* = 0.018	SS	0.001	0.037	0.025	*R^2^* = 0.024
CRF									
TSR	−0.002	−0.086	0.000	*F* = 7.308	NME	0.005	0.088	0.000	*F* = 4.116
PS	−0.001	−0.041	0.004	*R^2^* = 0.067	PS	−0.005	−0.092	0.000	*R^2^* = 0.032
SS	0.001	0.046	0.007		S-P	0.004	0.077	0.004	
					LCA	−0.003	−0.073	0.004	
					HA	0.003	0.053	0.030	
					PCR	0.002	0.045	0.043	
MSF									
PS	0.001	0.049	0.000	*F* = 6.761					
TSR	0.001	0.036	0.009	*R^2^* = 0.059					

Model 1 controlled for age. Model 2 further adjusted for CRF and MSF to BMI, for BMI and MSF to CRF, and BMI and CRF to MSF.
